# Case report: Epidermoid cyst misdiagnosed as a loculated pericardial effusion

**DOI:** 10.4103/0971-3026.40956

**Published:** 2008-11

**Authors:** NC Sharma, Anshu Sharma, Manish Bajaj

**Affiliations:** Department of Radiodiagnosis, R.N.T. Medical College and Associated Group of Hospitals, Udaipur (Rajasthan)-313 004, India

**Keywords:** Epidermoid, mediastinal, pericardial

## Abstract

A 25-year old man presented with a mediastinal lesion which was initially diagnosed as a loculated pericardial collection on echocardiography. Subsequent imaging showed it to be a cystic mediastinal mass, and following surgery and histopathology, it turned out to be an epidermoid cyst.

A mediastinal epidermoid cyst is extremely rare. It is characterized pathologically by keratinized epithelium that differentiates it from bronchoenteric, bronchopulmonary, and esophageal duplication cysts.[1] We report a patient with a large epidermoid cyst in the mediastinum that was mistaken for a loculated pericardial collection.

## Case Report

**Figure 1:**
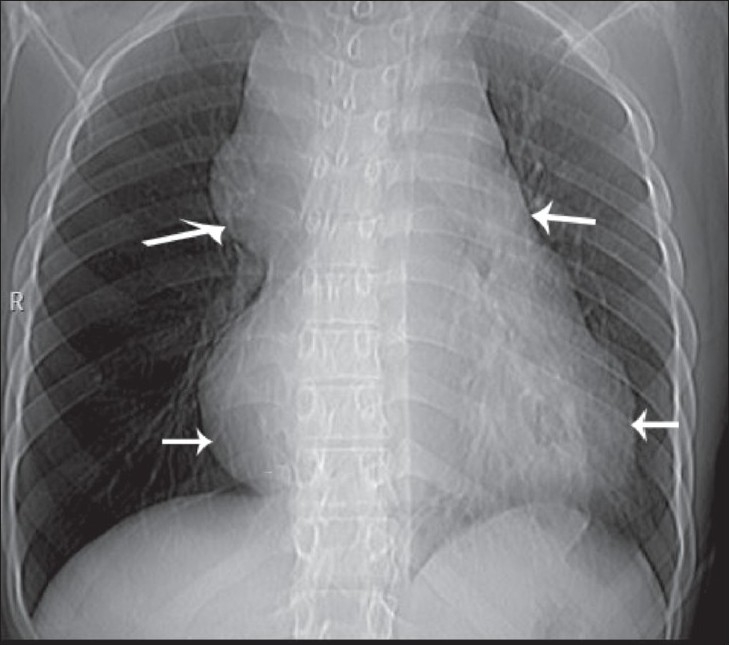
Frontal chest radiograph shows mediastinal widening (arrows)

A 25-year-old man presented to the emergency department with severe hypotension. There was a history of gradually progressive dyspnea over 6 months. Physical examination revealed engorged neck and chest wall veins and a swollen face. The jugular venous pressure was found to be raised, suggesting superior vena cava (SVC) compression. The frontal chest radiograph [Figure 1] showed mediastinal widening that was predominantly in the superior part.

**Figure 2 (A–C):**
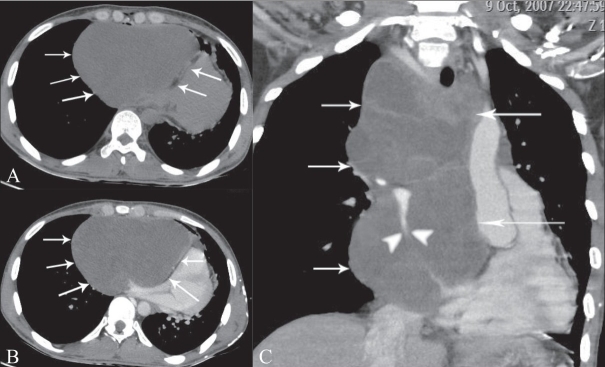
Axial, plain (A), post-contrast (B), and coronal post-contrast (C) images show a hypodense, non-enhancing lesion (arrows) displacing and compressing the heart as well as the superior vena cava (arrowhead)

**Figure 3:**
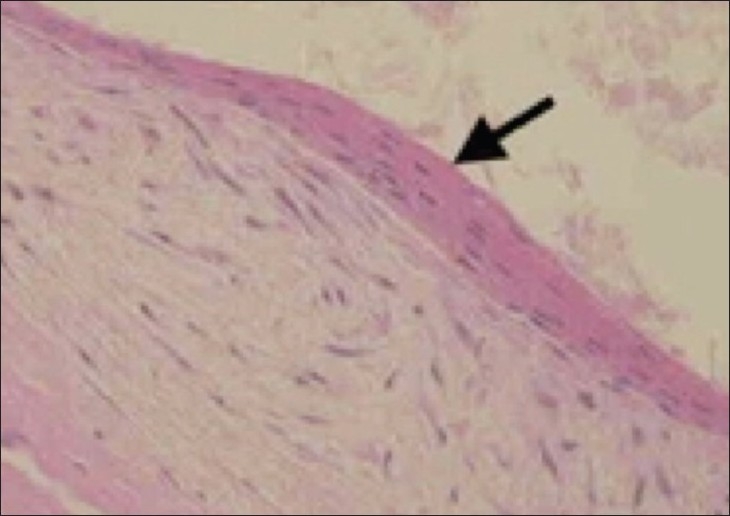
Histopathology section shows a fibrous wall lined by stratified squamous epithelium and covered with laminated keratin

Echocardiography was performed and a diagnosis of a loculated pericardial effusion on the right side of the heart was made; the heart had been displaced to the left and there was compression of the right atrium and ventricle. Pericardiocentesis yielded thick turbid fluid. CT scan of the chest showed a fluid-density cystic lesion with no enhancement; it was situated in the anterosuperior mediastinum and measured 183 × 132 × 154 mm in size. The lesion had displaced the heart to the left, with compression of the right atrium and ventricle; there was also encasement and compression of the major vessels, including the superior vena cava and its tributaries [Figure 2]. No associated vertebral anomaly was detected. The patient was taken up for immediate surgery, which revealed a large, tense, cystic mass in the anterosuperior mediastinum, attached to the pericardium. It was resected intact. There was no evidence of any intraspinal extension or fibrous connection with the thoracic spine. The cyst showed homogeneous contents and had a smooth wall. Histopathology revealed a fibrous wall lined by stratified squamous epithelium with laminated overlying keratin, consistent with a diagnosis of epidermoid cyst [Figure 3]. The patient unfortunately succumbed to postoperative respiratory complications

## Discussion

Dermoids and epidermoids are ectoderm-lined inclusion cysts, with dermoids showing skin appendages and epidermoids lacking them.[2]

Dermoid cysts appear as thin-walled, unilocular masses showing low Hounsfield numbers (0 to −18), suggesting the presence of fat.[2] Epidermoid cysts however show homogeneously decreased CT attenuation, appearing hypointense on T1W and hyperintense on T2W images, paralleling fluid signal intensities.[3] This appearance is indistinguishable from other mediastinal cystic lesions such as bronchoenteric, bronchopulmonary, and esophageal duplication cysts.

Esophageal duplication cysts appear as spherical or tubular masses in close proximity to the esophagus, sometimes adherent to the wall of the esophagus. They are usually homogeneous and exhibit water attenuation.[4,5] On CT, bronchogenic cysts appear as round or spherical, sharply marginated, homogeneous masses with water attenuation values in the majority of cases.[6] Increased CT attenuation may result from hemorrhage or proteinaceous debris within the lesion.[7]

The diagnosis of an epidermoid cyst can reliably be made only on histopathology. A typical epidermoid cyst is lined with stratified squamous epithelium that contains a granular layer and is filled with keratinous material that often has a laminated arrangement.

Posterior epidermoid cysts should be considered in the differential diagnosis of intradural extramedullary lesions of the spinal cord.[3] However anterior mediastinal epidermoid cysts, as in the present case, are very rare and there is no data available on their incidence. It is important to rule out any connection with the spine or spinal canal.

## References

[1] Sameh IS, Gewaeli NN, Hamza UA, Awadalla MM (2003). Epidermoid cyst radiologically mistaken as a left sided subpulmonic effusion. *Interact Cardiovasc Thorac Surg*.

[2] John C, John R Egelhoff pediatric head and neck imaging. CT and MR imaging of the Whole Body Haaga.

[3] Scarrow AM, Levy EI, Gerszten PC, Kulich SM, Chu CT, Welch WO (2001). Epidermoid cyst of the thoracic spine: Case history. Clin Neurol Neurosurg.

[4] Amstrong P, Wilson AG, Dee P, Hansell DM (1995). Imaging of diseases of the chest.

[5] Weiss LM, Fagelman D, Warhit JM (1983). CT demonstration of an esophageal duplication cyst. J Comput Assist Tomogr.

[6] Glazer HS, Siegel MJ, Sagel SS (1989). Low-attenuation mediastinal masses on CT. AJR Am J Roentgenol.

[7] Mendelson DS, Rose JS, Efremidis SC, Kirschner PA, Cohen BA (1983). Bronchogenic cysts with high CT numbers. AJR Am J Roentgenol.

